# Optimization of sunflower head pectin extraction by ammonium oxalate and the effect of drying conditions on properties

**DOI:** 10.1038/s41598-021-89886-x

**Published:** 2021-05-19

**Authors:** Xuemei Ma, Jiayi Yu, Jing Jing, Qian Zhao, Liyong Ren, Zhiyong Hu

**Affiliations:** School of Chemical Engineering and Technology, North University of China, Taiyuan, 030051 China

**Keywords:** Carbohydrates, Glycobiology

## Abstract

Pectin is a kind of natural and complex carbohydrates which is extensively used in food, chemical, cosmetic, and pharmaceutical industries. Fresh sunflower (*Helianthus annuus* L.) heads were utilized as a novel source of pectin extracted by ammonium oxalate. The conditions of the extraction process were optimized implementing the response surface methodology. Under optimal extraction parameters (extraction time 1.34 h, liquid–solid ratio 15:1 mL/g, ammonium oxalate concentration 0.76% (w/v)), the maximum experimental yield was 7.36%. The effect of spray-drying and freeze-drying on the physiochemical properties, structural characteristics, and antioxidant activities was investigated by FT-IR spectroscopy, high performance size exclusion chromatography, and X-ray diffraction. The results showed freeze-drying lead to decrease in galacturonic acid (GalA) content (76.2%), molecular weight (*M*_w_ 316 kDa), and crystallinity. The antioxidant activities of pectin were investigated utilizing the in-vitro DPPH and ABTS radical-scavenging systems. This study provided a novel and efficient extraction method of sunflower pectin, and confirmed that different drying processes had an effect on the structure and properties of pectin.

## Introduction

Pectin is a complex set of polysaccharides that is widely used as an additive in desserts, dairy products, and soft drinks as a gelling, stabilizing, and thickening agent^1^. Notably, low-methoxyl pectin (LMP) can form gel without sugar. LWP can be used as a fat substitute and a low-calorie product^2^, which is particularly suitable to being included in the diet of people with obesity. However, commercial LMP is generally prepared from high-methoxyl pectin (HMP), which renders the production cost of LMP higher^3^. The previous studies proved that sunflower heads were rich in natural LMP (15–25%)^4^, which not only expanded the application scope of waste sunflower heads, but also enriched the structural characteristics of pectin^5^.

Conventional methods of extracting sunflower pectin generally rely on dry sunflower heads^4,6,7^ and require harsh acid and high liquid–solid ratio (25:1–60:1)^6,8^. Such a way could certainly lead to acidic degradation of pectin, the large amount waste of ethanol and serious environment pollution due to the acidic solid waste^9^. In this context, ammonium oxalate and sodium hexametaphosphate solutions have been observed to be effective in the extraction of pectin from sunflower heads; in fact, the pectin obtained by the latter had a higher ash content^10^. Through the direct reaction of ammonium oxalate with pectin, acid pollution may be reduced, and the formation of hydrogen bonds between pectin chains may be enhanced which endow pectin with diverse structural features^3^. On the other hand, during the drying process of fresh sunflower heads, polysaccharides and proteins undergo a complex browning driven by oxygen and higher temperature to produce a substance called melanin pigment, whose presence influences the sensory quality of pectin^12^. However, the industry generally prefers lighter colored pectin^11^. Therefore, a variety of decolorization processes are used in the extraction process of commercial pectin to obtain qualified pectin, including activated carbonin decolorization, alcohol ammonia solution decolorization, etc^12^. These approaches caused a decrease in pectin yield, expensive operation, and destroyed pectin molecules structure^13^. Therefore, extracting fresh sunflower heads to obtain LMP has economic and environmental significance. In previous studies, fresh sunflower heads were extracted by subcritical water to obtain LMP with ideal color by subcritical water extraction^14^, but the obtained pectin had low *M*_w_.

Muthusamy et al.^15^ based on response surface method of genetic algorithm and artificial neural network model to extracted dry sunflower heads, and the pectin yield was 29.5%. The use of RSM can increase product yield and reduce process differences, thereby shortening development time and reducing overall cost. Some studies indicated drying methods had an effect on physiochemical properties^11^ and various function of pectin, such as antioxidant activity^19,20^ and emulsifying properties^21^. Antioxidant property of pectin was the most widely studied^16,17^. Researches have proved the pectin-polyphenol conjugate have improved antioxidant properties with respect to polyphenol^18^. Among the different drying methods, spray-drying and freeze-drying are the most common ways to convert liquid products for powders with high chemical and biological stability^22^. Up to date, there is no study on the effect of drying methods on physiochemical properties of pectin from pectin from fresh sunflower heads (SFHP).

In order to obtain pectin with rich structure, qualified quality and high yield, in this study, ammonium oxalate was used to extract pectin from fresh sunflower heads. The RSM was to apply to optimize the operating parameters. Pectin was obtained under the optimized conditions and then dried by two different methods (freeze-drying and spray-drying). The relationship between drying treatments and pectin properties (physiochemical properties and antioxidant activities) was investigated.

## Results and discussion

### The results of single factor analysis

**Figure 1:**
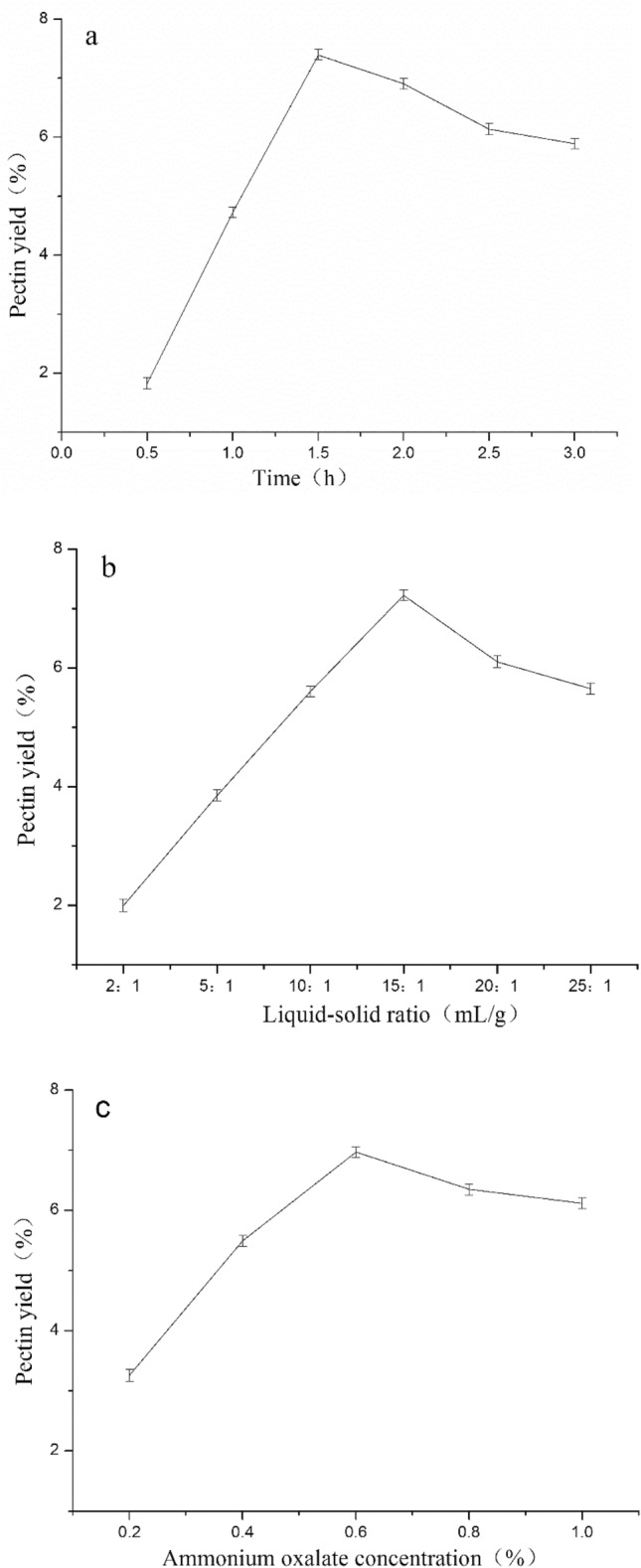
Single factor analysis showing the effect of each independent variable (**a** time, **b** liquid–solid, **c** ammonium oxalate) on the yield of the SFHP.

The influence that various factors had on pectin yield is reflected by the data reported in Fig. 1. To determine the effect of extraction time on pectin yield, the extraction was performed for 0.5–3 h, with the liquid–solid ratio of 15:1 mL/g, and the ammonium oxalate concentration at 0.4% (w/v) at 85 °C (Fig. 1a). The pectin yields initially increased as the extraction time increased, reaching the maximum yield (7.9%) at 1.5 h. Then, the yields started to decrease, which may due to prolonged extraction time decomposed the pectin, and separation was more difficult^23^. Therefore, 1.5 h was selected in the subsequent investigations.

The effect of liquid–solid ratio (2:1–25:1 mL/g) on the pectin yield was studied by fixing the temperature at 85 °C, the ammonium oxalate concentration at 0.4% (w/v) and the extraction time for 1.5 h. As can be evinced from the data in Fig. 1b, the yield of pectin significantly increased as the liquid–solid ratio increased from 2:1 to 15:1 mL/g, whereas it decreased after 15:1 mL/g. This observed trend may be related to the sufficient solvency of the target compounds in a larger volume of extraction solvent, as has been reported by previous result^24^. However, the concentration of solute was reduced at higher liquid–solid ratio (> 15:1 mL/g), resulting in an increasing driving force for diffusion and dissolution^25^. In the subsequent experiments, the liquid–solid ratio was restricted to 15:1 mL/g, which was significantly lower than that reported for previously described processes of pectin extraction from sunflower heads.

The impact of the ammonium oxalate concentration on the yield of pectin was achieved under the conditions where the temperature, liquid–solid ratio and extraction time were set to 85 °C, 15:1 mL/g and 1.5 h, respectively. The data in Fig. 1c revealed that the extraction yield of pectin initially increased alongside the ammonium oxalate concentration and reached the peak value (9.2%) when the ammonium oxalate concentration was 0.6% (w/v), then appeared to reach a plateau. The mechanism of the ammonium oxalate extraction method is to turn insoluble calcium pectate into soluble ammonium salt^26^. 0.6% (w/v) ammonium oxalate had maximized pectin hydrolysis. Therefore, 0.6% (w/v) was selected as optimum.

All in all, the optimal conditions for pectin extraction were thus concluded to be the following: 85 °C, 1.5 h extraction time, 15:1 mL/g liquid–solid ratio, and 0.6% (w/v) ammonium oxalate concentration. These values were utilized for subsequent experiments using the Box–Behnken Design (BBD).

### Optimization of the extraction parameters by RSM

As a collection of mathematical and statistical technique, the BBD could examine the factors (A: extraction time, B: liquid–solid ratio, and C: ammonium oxalate concentration) effect on responses (pectin extraction yield) and it was applied to process optimization^24^. Table 1 showed the coded and true value of the extraction variables, the model predicted, and experimentally determined response. According to the data in this table, pectin extraction yield ranged between 1.03 and 7.86%.

**Table 1:** ANOVA for the proposed model of SFHP extraction yield.

Source of variance	DF	Some of square	Mean square	*F*-value	*P* value
Model	9	71.32	7.92	66.76	< 0.0001
A	1	10.83	10.83	91.28	< 0.0001
B	1	19.69	19.69	165.87	< 0.0001
C	1	14.10	14.10	118.78	< 0.0001
AB	1	2.19	2.19	18.45	0.0036
AC	1	3.35	3.35	27.45	0.0012
BC	1	0.53	0.53	4.43	0.0734
A^2^	1	3.62	3.62	30.48	0.0009
B^2^	1	11.84	11.84	99.77	< 0.0001
C^2^	1	3.33	3.33	28.07	0.0011
Residual	7	0.83	0.12	–	–
Lack-of-Fit	3	0.14	0.046	0.27	0.8477
Pure error	4	0.69	0.17		
Total	16	72.15			
R^2^		0.9885			
Adj R^2^		0.9737			
Pred R^2^		0.9544			

ANOVA was performed to check the predictive nature of the model, the significance level of each variable and interaction effects^23^. As shown in Table 1, The F-value and *P*-value of lack-of-fit in the regression model were 0.27 and 0.8477, respectively, which indicated that the lack-of-fit was insignificantly relative to the pure error and confirmed the validity of the model^27^. In the regression model, the *R*^2^ values of 0.9885 indicated that the fitted model can explain 98.85% of the variations. The adjusted determination coefficients (*R*_Adj_^2^ = 0.9737) values also confirmed that the model was acceptable in terms of experimental errors and reliable for the prediction of the experimental yield. The order of the effect of three independent variables was B > C > A, which indicated that liquid–solid ratio (B) had the largest effect (*P* < 0.0001) on SFHP yield. The interaction terms of extraction time with liquid–solid ratio (AB) and extraction time with ratio of liquid–solid (AC) also showed significant effects (*P* < 0.05) on SFHP yield. Multiple regression analysis was performed on the experimentally obtained data using the Design-Expert software, and a second-order polynomial equation (Eq. 1) with significant fit (*P* < 0.0001) and non-significant lack-of-fit (*P* > 0.05) was successfully constructed to express the interactions effect of the extraction variables and the response.

1 $$\begin{aligned} {\text{Y}}\left( \% \right) & = 6.16 + 1.16A + 1.57{\text{B}} + 1.33{\text{C}} + 0.74{\text{AB}} \\ & \quad + 0.90{\text{AC}} + 0.36{\text{BC}} - 0.93{\text{A}}^{2} - 1.68{\text{B}}^{2} - 0.89{\text{C}}^{2} \\ \end{aligned}$$

where Y is the SFHP yield (%), and A, B, and C are the coded values of the extraction time (h), and liquid–solid ratio (mL/g), and ammonium oxalate concentration (%), respectively.

**Figure 2:**
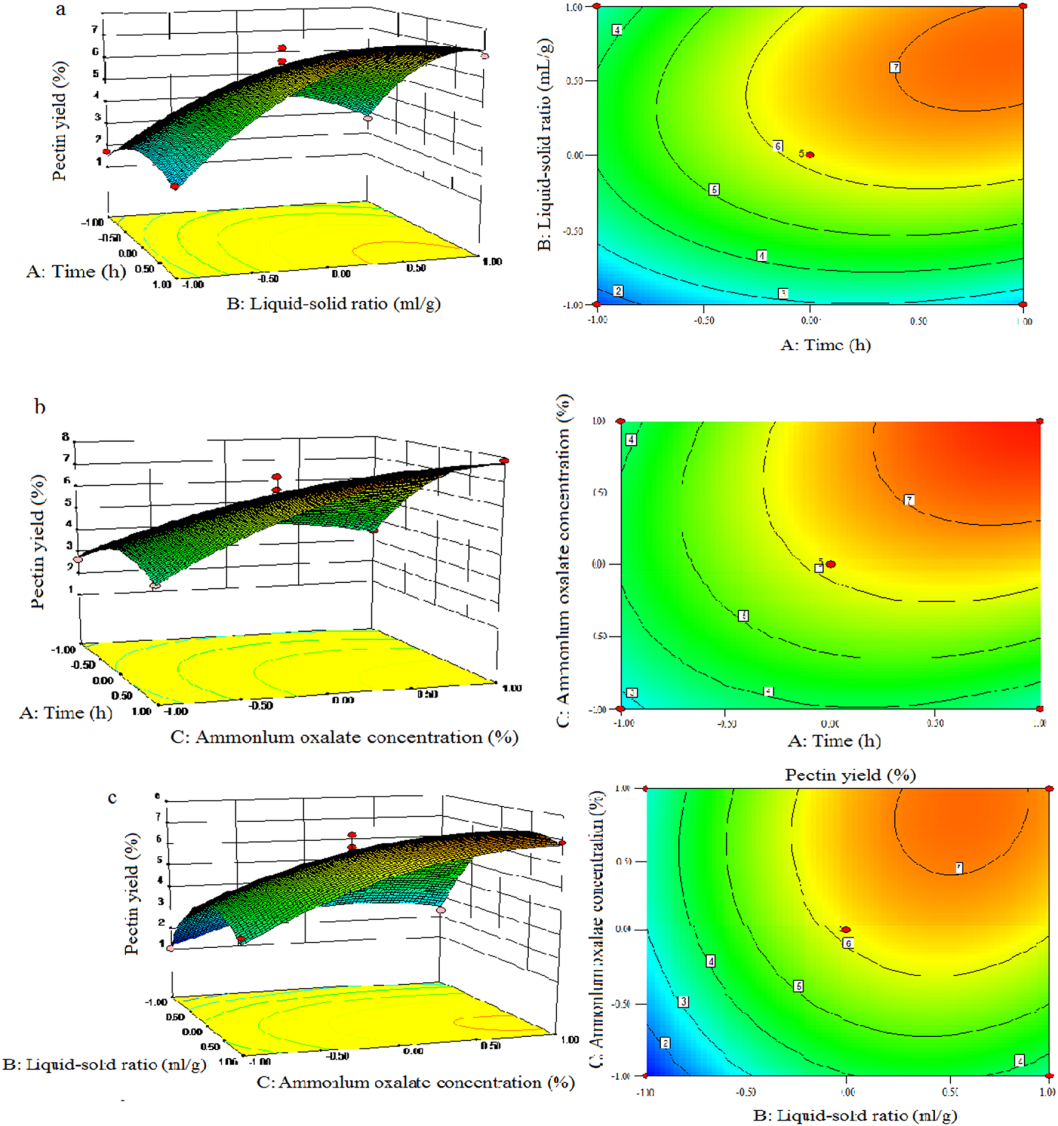
Response surface plots shows the significant mutual effects of extraction time (**A**), liquid–solid ratio (**B**), and ammonium oxalate concentration (**C**) on the yield of SFHP.

Figure 2 showed a graphical description of the two-dimensional (2D) contour and three-dimensional (3D) response surface maps generated by the software, which represented the influence of each factor on the predicted value, and the interaction between two arbitrary variables^23^.

Figure 2 illustrated that an increase in the value of each independent variable in the studied range can significantly increase the extraction yield. The effect of extraction time on pectin yield was investigated and results showed in Fig. 2a, b. The mentioned figures indicated that as the extraction time increased, so did the pectin yield. Meanwhile, the contours of 2D contour plot were elliptical, indicating that a significant interaction existed between the two dependent variables (AB, AC). As indicated by the data reported in Fig. 2c, the contour plot of the interaction between B and C was circular, which mean the interaction between B and C was not significant.

Analysis using Design-Expert 9.0 showed that the set of maximum predicted values for the optimal pectin yield was as follows: extraction time 1.34 h, liquid–solid ratio 15:1 mL/g, ammonium oxalate concentration 0.76% (w/v) and the predicted maximum extraction rate 7.86 (g/100 g). In order to ensure that the predicted value did not deviate from the real experimental value, three extractions were performed using the predicted optimal extraction conditions, and 7.36 ± 0.4 (g/100 g) was obtained. This value was in good agreement with the value predicted by the model, indicating that the model can be safely used to optimize the extraction of pectin. It is worth noting that the yield of fresh sunflower pectin extracted by ammonium oxalate is higher than that of pectin extracted by subcritical water^14^.

### Effect of the raw materials on the pectin color

The color of pectin is a key quality parameter used in food and biological preparations^20^. The effect of raw material on color coordinates (L^*^, a^*^, b^*^, H^*^_ab_, and C^*^), and total color difference (ΔE) were recorded in Table 2. The L^*^ value represents the lightness of the color, ranging from 0 (black) to + 100 (white), while the a^*^ value ranges from − 100 (green) to + 100 (red), and the b^*^ value ranges from − 100 (blue) to + 100 (yellow)^9^. Compared with pectin from pectin from dry sunflower heads (DSHP), SFHP displayed a higher value for lightness (L^*^); SFHP also displayed similar brightness to sigma commercial pectin. The ΔE value of DSHP was 30.83, indicating that the color of pectin from different raw material was significantly different (30.83 > 6.77), so color changes were visible to the naked eye^20^.

**Table 2:** Color parameters (L^*^, a^*^, b^*^, ΔE, H^*^_ab_, C^*^ (lightness, redness, yellowness, total color difference, hue angle, and chroma, respectively)) and visual aspect (pictures) of different pectin power.

	Sigma commercial pectin	SFHP	DSHP
Visual aspect			
L*	81.23 ± 0.25	75.88 ± 0.38	51.99 ± 0.52
a*	4.01 ± 0.07	4.14 ± 0.13	2.86 ± 0.24
b*	14.88 ± 0.17	10.73 ± 0.20	5.17 ± 0.38
H*_ab_	15.06 ± 0.12	21.11 ± 0.17	28.94 ± 0.42
C*	15.41 ± 0.18	11.50 ± 0.23	5.91 ± 0.45
ΔE*		6.77 ± 0.21	30.83 ± 0.48

In fact, during the drying process of sunflower heads, various phenols were prone to browning or caramel browning^12^ and produced the oxidation products of colored phenolic compounds (OXP)^28^. In the other hand, the melanin produced by the oxidation of polysaccharides and proteins is water soluble and is tightly associated with the pectin extract, resulting in poor quality of pectin from dried sunflower heads. Therefore, pectin extracted from fresh sunflower heads by ammonium oxalate not only avoids the production of undesirable pigments, but also weakens the bonds between pectin and water-soluble colorants, effectively causing pectin to display a lighter color^12^.

### Physicochemical properties of spray-dried pectin (SP) and freeze-dried pectin (FP)

#### Molecular weight

Chemical composition and weight distribution of SP and FP were shown in Table 3. For pectin, the rheological behaviors and gelling properties significantly depend on the *M*_w_^3^. The *M*_w_ of FP (316 ± 3 kDa) was found to be slightly lower than that of SP (336 ± 3 kDa). However, no matter which drying method was used for drying pectin, the *M*_w_ of SFHP extracted by ammonium oxalate was larger than that of sunflower pectin extracted by ultrasound-assisted method^46^ or subcritical water method^14^.

**Table 3:** Chemical and physicochemical properties of FP and SP extracted under optimal extraction conditions.

Chemical composition	FP	SP
DE (%)	36.4 ± 0.2	39.2 ± 0.3
GalA content (% w/w)	76.2 ± 0.1	85.9 ± 0.2
Ash content (% w/w)	2.1 ± 0.2	1.9 ± 0.1
Es_1_ (% w/w)	75.3 ± 0.4	88.9 ± 0.2
Es_5_ (% w/w)	49.4 ± 0.3	71.6 ± 0.1
pH	2.68	2.76
WHC (% w/w)	91.4 ± 0.5	73.6 ± 0.6
**Molecular weight distribution**		
*M*_n_ (kDa)	301 ± 4	330 ± 2
*M*_w_ (kDa)	316 ± 3	336 ± 3
*M*_w_/*M*_n_	1.05	1.01

#### Degree of methylesterification (DE)

**Figure 3:**
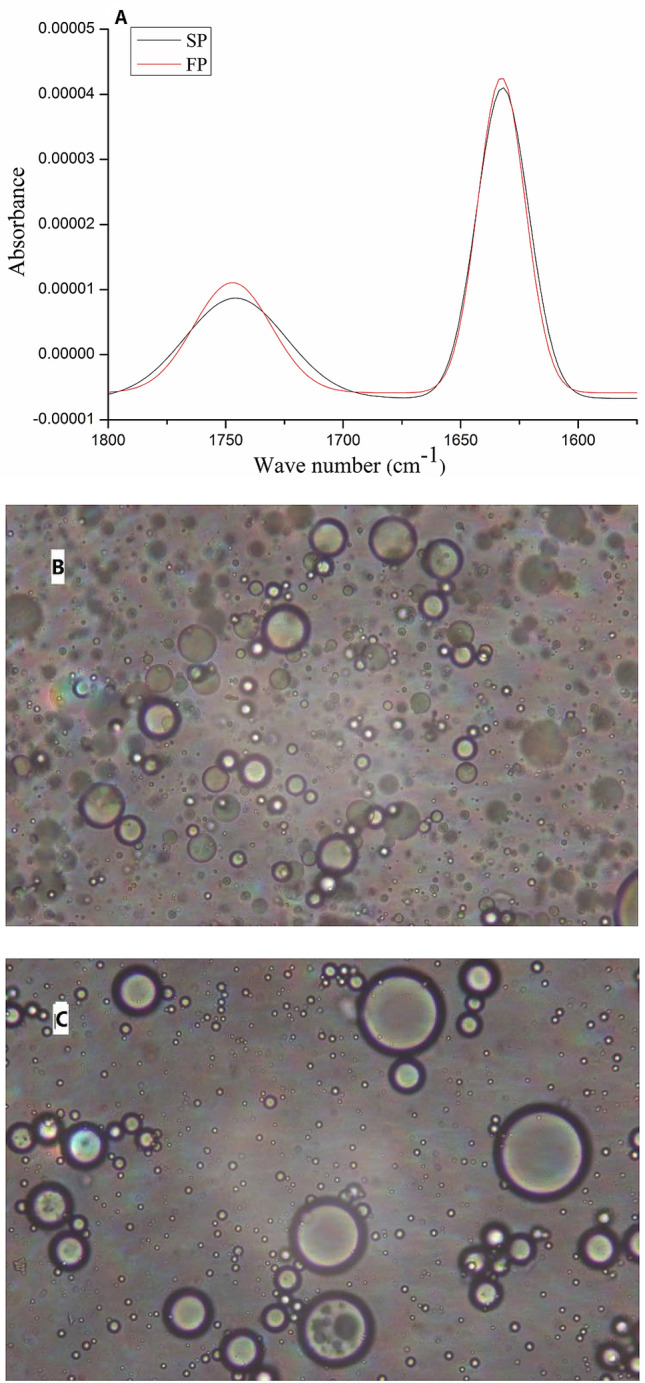
Deconvoluted spectral region of FP and SP (**A**); microscopic images of FP (**B**) and SP (**C**).

For DE analysis, the deconvoluted spectra (1600–1800 cm^−1^) of SP and FP was shown on Fig. 3, where the bands at 1745–1750 and 1616–1634 cm^−1^ were respectively assigned to stretching vibrations of the esterified and ionized carboxyl groups of the pectin molecules^29^. Therefore, the DE value of SP and FP were calculated to be 39.2 ± 0.3% and 36.4 ± 0.2%, respectively, which indicated that the SP and FP should be categorized as LMP^30^. Results from a previous study^19^ pointed that the intensity of the absorbance or band area of the ester carbonyl groups (1730–1760 cm^−1^) increased with the increase in DE, while the intensity of the absorbance or band area of the free carboxylate groups (1650–1600 cm^−1^) increased as the DE value decreased.

#### GalA content

According to recommendations of FAO^31^ (Food and Agriculture Organization) and European Union, the GalA content of pectin used as a food additive or pharmaceutical purpose should not be lower than 65%^32^, and other research pointed the GalA content of pectin generally varied from 56 to 72.6%^33^. FP (76.2%) displayed a lower GalA content than SP (85.9%), even though both samples had been extracted in the same conditions, which emphasized the importance of the drying method with respect to the GalA content in the extracted pectin. Notably, the ash contents were similar between SP and FP.

#### Emulsifying properties

In the emulsification process, short time stability was evaluated by centrifugation which reflected the ability of droplets to resist recoalescence^34^. As shown in Table 3, emulsions from SP showed better stability. Furthermore, SP exhibited extremely good centrifugation stability reaching 88.9% for ES_1_ and 71.6% for ES_5_. The difference may be due to freeze-drying caused degradation of pectin. In addition, some studies^35^ have pointed out that the more hydrophobic groups in pectin, the higher its emulsifying ability. Pectin with a high degree of esterification had good emulsifying properties, which was consistent with the previous DE results.

The optical microscopy images of FP and SP were presented in Fig. 3. The emulsion formed with SP (Fig. 3C) showing larger droplet size than that formed with FP (Fig. 3B). Previous study^37^ pointed out that the size of the droplets determined the emulsification activity and can indicate the stability of the emulsion. The unadsorbed SP with high *M*_w_ led to an increased viscosity of the aqueous phase, which limited the droplet movement and preventing further flocculation or coalescence^34^.

#### Water-holding capacity (WHC)

Under the same conditions, FP (91.4%) was easier to form gel and retained more water than SP (73.6%). Pectin gelation was formed by the calcium bridges which needed a certain proportion of dissociated carboxyl groups, and adding a small amount of sucrose can avoid syneresis of the gel^37^. It has been reported that WHC may be due to the degradation of neutral sugar side chains in the hairy regions of pectin, which led to higher charge density and more opportunities to form ion junction regions^36^. Freeze-drying may caused degradation of pectin side chains, resulting in the more charge exposed, the easier ion junction region formed.

### FT-IR analysis of SP and FP

**Figure 4:**
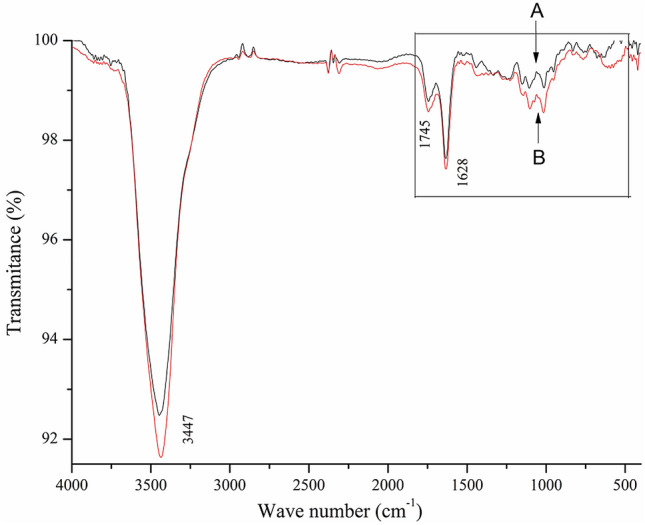
FT-IR spectra of FP (**A**) and SP (**B**).

In order to test the effect of drying method to the structure on pectin, FT-IR spectrometry data were collected (Fig. 4). The obvious absorption peak at 3447 cm^−1^ was caused by the stretching vibrations of O–H bound, and the stretching vibration of the C–H bond was represented by weak peak at 2924 cm^−1^^20^. It can be observed that there was slightly different about the intensity of the absorption peaks at 1745 and 1628 cm^−1^ which ascribed to C=O stretching vibrations of the esterified carboxyl group (COOR) and ionized carboxyl group (COO^−^) respectively. The DE value has been reported to generally reflected in proportion to the 1744 cm^−1^ peak. Hence, the characteristic absorption led to a speculation that the spray drying of the pectin decreased the DE value. The fingerprint region (1350–400 cm^−1^) may reflect some changes in the composition of the pectin monosaccharide. The absorption peaks around 1107 and 1016 cm^−1^ indicated that the sample contained glycosidic bonds and pyranoid rings, respectively^38^. In addition, the band around 609 cm^−1^ was attributed to C–C stretching vibration of pyranoid rings. The absorption of FP had an obviously decrease at 1107 cm^−1^, 1016 cm^−1^ and 609 cm^−1^^39^, which illustrated that the covalent bounds of pyranose monosaccharide were probably destroyed by the freeze-drying. Both pectin had very similar characteristic absorption peak position.

### Crystallinity of SP and FP

**Figure 5:**
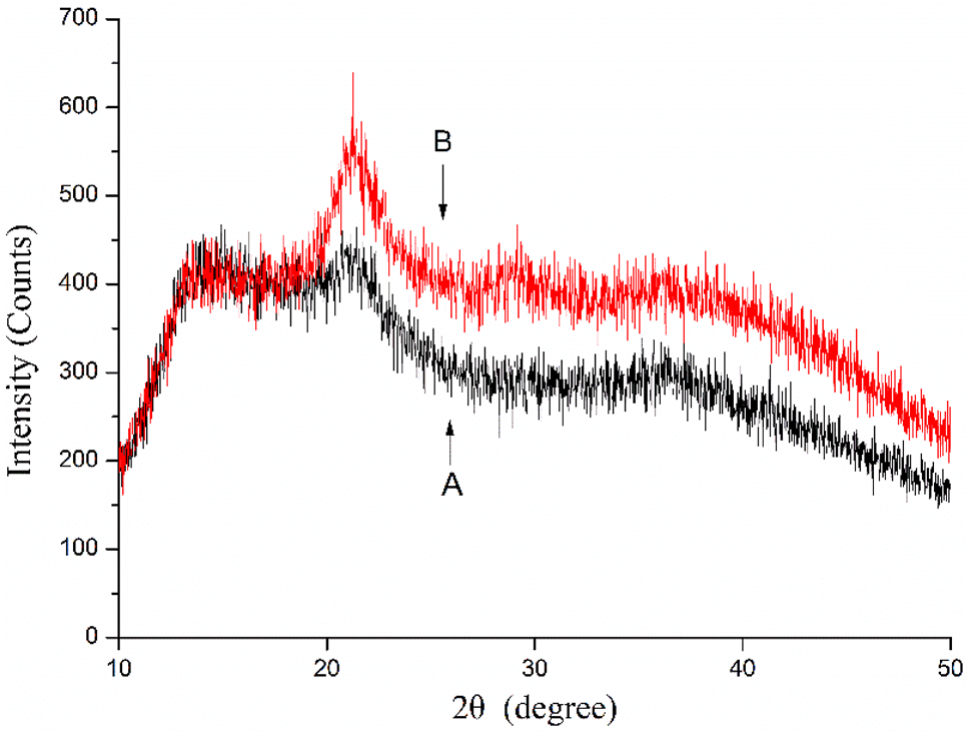
XRD of the FP (**A**) and SP (**B**).

XRD was used to provide more structure information on the pectin (amorphous or crystalline)^40^. The XRD patterns of SP and FP were reported in the Fig. 5. The crystallinity of FP and SP was almost similar and mainly consisted of amorphous nature. However, the peak intensity for SP was slightly higher than for FP and several sharp and intense peaks as illustrated at 21.29°, 29.12°, and 38.22° (2θ) of SP. This evidence might indicate that the crystallinity of some small portions in SP structure. According to the previous research, spray-drying could lead to better mechanical performance^41^. Some researchers illustrated that the decrease in molecular weight and the changes of physical structure resulted in the variation in XRD patterns^40^. Therefore, it can be noted that extraction by ammonium oxalate and freeze-drying treatment changed the physical structure and the crystallinity of pectin.

### Radical-scavenging ability of SP and FP

#### DPPH radical scavenging of pectin

**Figure 6:**
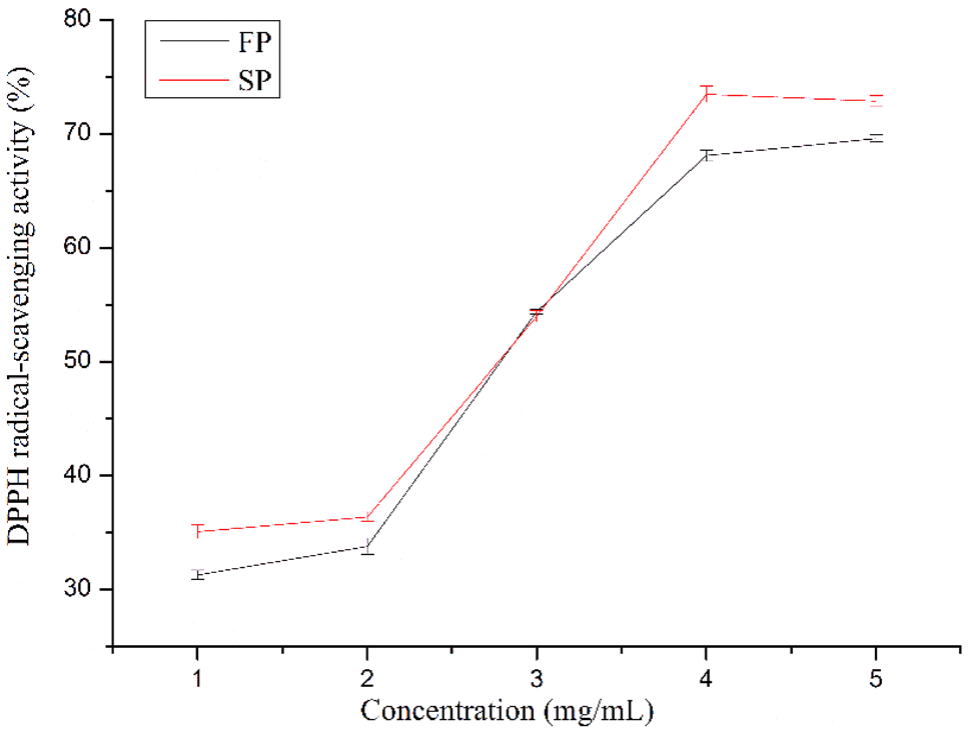
Effects of pectin on DPPH radical-scavenging activity.

The DPPH radical scavenging, as one of the most practical tests for determination of the antioxidant activity, was applied to estimate the antioxidant activity of SP and FP. As shown in Fig. 6, with increasing concentration (1–4 mg/mL), the DPPH free radical-scavenging activities of SP and FP were positively correlated with concentrations; moreover, the removal rate of SP was larger than FP. For polysaccharides, the transfer of the hydroxyl groups and electrons from pectin (ROH or RO^−^) to DPPH radicals is the main mechanism for the termination of the free radical chain reaction and the scavenging of DPPH radicals. Previous reports pointed that a relatively low molecular weight and a high GalA content in pectin appeared to be associated with high antioxidant activity^42^, a general trend that is consistent with the HPSEC and GalA content results on SP and FP. With the previously reported^27,43^, this result could be due to the freeze-drying treatment reduced the GalA content.

#### ABTS radical-scavenging activity of pectin

**Figure 7:**
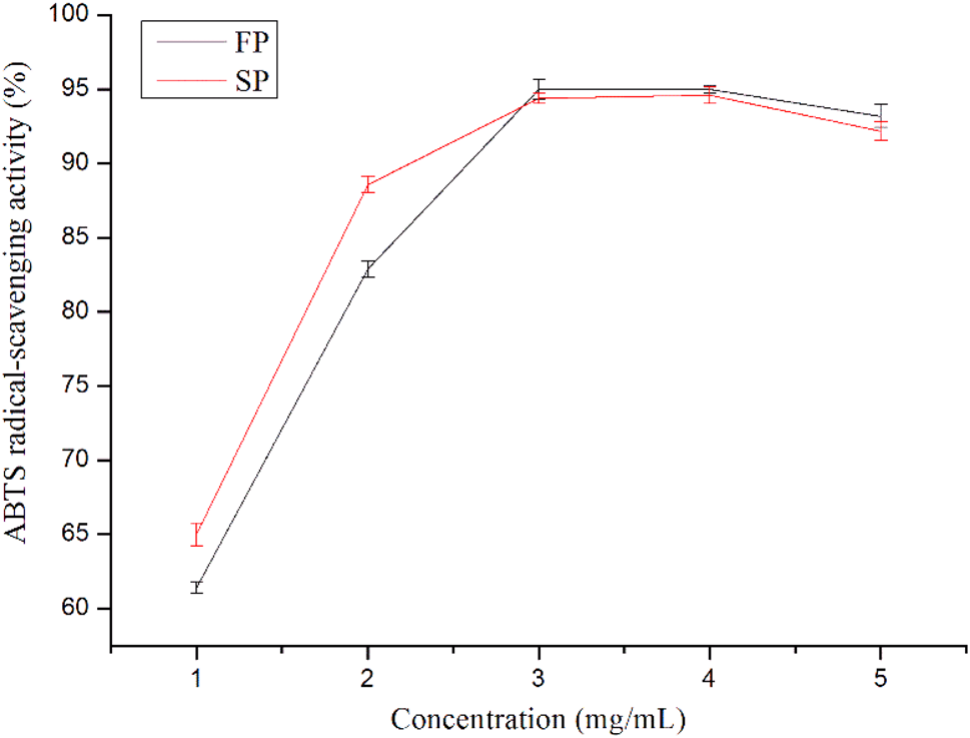
Effects of pectin on ABTS radical-scavenging activity.

In order to further verify the antioxidant activities of the SP and FP, the ability of SP and FP to scavenge ABTS free radicals was compared (as shown in Fig. 7). The scavenging activity of SP and FP on ABTS free radicals significantly increased as the concentration of samples increased from 1 to 3 mg/mL, then decreased at 3 mg/mL. The removal rate of SP was larger than FP when the concentration is between 1 to 3 mg/mL. The antioxidant activity of polysaccharides has been reported to be considerably affected by *M*_w_, conformation, and monosaccharide composition^27^. In other words, the observe could be related to that hydrolysis of FP reduced proton donation from hydroxyl and uronyl group of the monosaccharide units, carboxyl group of galacturonic acid units and acetyl groups^16^.

## Materials and methods

### Raw materials and regents

Fresh sunflower heads (water content 72.5–83%) were collected from Taiyuan, China. Ammonium oxalate, ethanol (99.8%) and acetonitrile (HPLC grade) were provided by Kaitong (Tianjin, China). Sodium nitrate and potassium bromide were provided by Damao (Tianjin, China). d-Galacturonic acid, trifluoroacetic acid, sodium hydroxide, hydrochloric acid, and phosphoric acid were provided by Aladdin (Shanghai, China). Commercial apple pectin (93,854) purchased from Sigma-Aldrich (Shanghai, China).

### Extraction of sunflower heads pectin

According to GB25533-2010^44^, the pretreatment method of fresh sunflower heads was developed by Ma et al.^14^*.* The fresh sunflower heads blocks were weighed with electronic scale (YP-10001, Shengke Instrument Equipment Co., Shanghai, China) and boiled in a constant temperature (above 95 °C) for 15–20 min, then filtered with 100 mesh gauze. High temperature can damage the structure of the pectin and lead to degradation. Therefore, 85 °C was selected in the subsequent investigations. Ammonium oxalate solution was added to filter cake under the temperature at 85 °C. Subsequently, the extracted liquid was concentrated and mixed with ethanol (95%) at 1:3 to prompt pectin precipitate, and the obtained mixture was equilibrated for 24 h. In the next step, the supernatant was separated by centrifugation (1000×*g*, 15 min, GT16-3, Era Beili Centrifuge Co., Beijing, China), then washed with ethanol (three times) and filter with 300–500 mesh nylon cloth to obtain pectin. After purification, the wet pectin was dried by electric heating blast drying oven (DHG-9023A, Yiheng Instrument Science Co., Ltd., Shanghai, China). The pectin yield can be expressed as Eq. (2):

2 $${\text{Pectin}} (\% ) = \frac{{{\text{m}}_{1} }}{{{\text{m}}_{2} }} \times 100$$

where m_1_ is the mass of pectin; m_2_ is the mass of flesh sunflower heads blocks.

### Single-factor experiment

The liquid–solid ratio (2:1, 5:1, 10:1, 15:1, 20:1, 25:1 mL/g), concentration of ammonium oxalate (0.2%, 0.4%, 0.6%, 0.8%, 1.0%), and extraction time (0.5, 1.0, 1.5, 2.0, 2.5, 3.0 h) on the extraction rate of pectin was investigated. All experiments were conducted in triplicate.

### Response surface methodology

Based on the single-factor experiment, the best value of each factor was obtained when the pectin yield is the highest. The BBD (Table 4) with 3 factors (concentration of ammonium oxalate, liquid–solid ratio, and extraction time) and a three-level response surface were implemented to optimize the extraction of SFHP. Using random combinations of independent variables to estimate experimental error, a total of 17 experiments were generated, including 5 center points and 12 factor points. The Design Expert 9.0 software was employed to analyze the result through maintaining two variables at central levels and constructing 3D plots of two factors.

**Table 4:** Box–Behnken Design matrix with measured and predicted values.

Run	Extraction time (h)	Liquid–solid ratio (mL/g)	Mass fraction of ammonium oxalate (%)	Experimental pectin yield (%)	Predicted pectin yield (%)
1	1.5	15:1	0.6	5.67	6.16
2	2.0	15:1	0.8	7.86	7.74
3	1.0	15:1	0.8	3.47	3.44
4	1.5	20:1	0.8	6.89	6.78
5	1.5	10:1	0.4	1.03	1.14
6	1.5	15:1	0.6	5.76	6.11
7	1.5	15:1	0.6	6.04	6.11
8	1.5	15:1	0.6	6.27	6.11
9	1.0	10:1	0.6	1.72	1.48
10	1.5	10:1	0.8	2.29	2.99
11	1.0	20:1	0.6	2.69	3.23
12	2.0	10:1	0.6	2.31	2.17
13	2.0	15:1	0.4	3.42	3.45
14	1.5	15:1	0.6	6.83	6.11
15	1.0	15:1	0.4	2.64	2.75
16	2.0	10:1	0.6	6.88	7.14
17	1.5	10:1	0.4	3.49	3.22

### Color measurement

The color of pectin sample was measured by a colorimeter (SC-10, 3nh, Guangdong Province, China). The colors of pectin samples extracted from dry and fresh sunflower heads under the optimal extraction conditions were compared with the color of sigma commercial pectin.

## Drying of pectin

After filtering and washing, the SFHP extracted by the optimal condition was dried by freeze-drying and spray-drying respectively. FP and SP were ultimately obtained.

### Freeze drying

A freeze dryer (CTFD-10P, Yonghe Chuangxin Electronic Technology Co., Ltd., Qingdao, China) was used to freeze dry the pectin solution for 8 h with the temperature ranged from − 30 to 20°C^45^.

### Spray drying

The filtered pectin was dried with a desktop spray dryer (YC-015, Pilotech, Shanghai, China). The spray conditions were as follows: the inlet temperature was 180 °C, the outlet temperature was 70 °C and the feed rate was 18 mL/min^7^.

## Physicochemical and structural characteristics of FP and SP

### Molecular weight distribution

The *M*_w_, number-average molecular weight (*M*_n_), and distribution of SP and FP samples were determined by HPSEC combined ACQUITY APC columns with ACQUITY^®^ Advanced Polymer Chromatography™ (APC™) system (Waters Technology Co., Shanghai, China). Different molecular weight (2.8, 20.4, 62.9, 111.9, 212.5, 310.2, and 390 kDa) of dextran standards was used as a calibration curve.

### GalA content

The GalA content of pectin was determined by method of Ezzat et al^46^. Released GalA was derivatized using 1-phenyl-3-methyl-5-pyrazolone (Karamar Ziyi Reagent Factory, Shanghai, China) and the derivatives were analyzed by RIGOL L3000 HPLC (Waters Corporation, Milford, MA, USA) with a Kromasil C18 column (250 mm × 4.6 mm, 5 μm) (Akzo Nobel Company, Sweden) and using d-galacturonic acid (> 97%, Aladdin, Shanghai, China) as standard.

### Ash content

According to the method of Kazemi et al*.*^43^ the ash content of the extracted pectin was determined by incinerating 1 g of pectin in furnace at 550 °C for 6 h.

### Emulsion stability

1% pectin solutions and corn oil (9:1 w/w) were mixed to prepare an emulsion. The mixtures were homogenized with a high-speed dispersion machiner (XHF-DY, Scientz Biological Technology Co., Ltd., China) at 3000 rpm for 3 times (1 min each time). The short-term stability of the prepared emulsion was measured by centrifugation. The emulsion was centrifuged for 1 min and 5 min with a high-speed centrifuge at a speed of 3000 rpm and a temperature of 20 °C respectively^34^. The height of the emulsified layer was recorded to calculate the emulsification stability of the pectin emulsion (Eq. 3).

3 $$\begin{aligned} {\text{Es}}_{{1}} (\% ) & = \frac{{{\text{H}}_{1} }}{{{\text{H}}_{{0}} }} \times 100 \\ {\text{Es}}_{{5}} (\% ) & = \frac{{{\text{H}}_{5} }}{{{\text{H}}_{{0}} }} \times 100 \\ \end{aligned}$$

where H_0_ is the height of emulsified layer without centrifugation; H_1_ and H_5_ are the height of emulsified layer centrifuged for 1 min and 5 min respectively.

### Light microscopy

The microstructures of the prepared emulsions were photographed immediately after the preparation by using a 40× objective lens on a BK6000 inverted microscope (Optec Co. Ltd., Chongqing, China), equipped with a digital microscope image analysis and processing software.

### Water-holding capacity (WHC)

The preparation of pectin gel was made according to the method of Wan^47^ with slight modifications. Pectin (1%, w/v) was dissolved in water. The pH was measured adjusted to 4.0 with 1 M NaOH, then sucrose (20 g/100 mL) and CaCl_2_ (40 mg/g, Ca^2+^/Pectin) were added under heating and stirring. A certain initial mass (W_1_) of gel system was add to centrifuge tube, and centrifuged at 4000 rpm for 20 min. After exudative water was drained, the gel mixture was weighted (W_2_). All the samples were measured in triplicate. The WHC can be expressed as Eq. (4).

4 $${\text{WHC (\% )}} = \frac{{{\text{W}}_{2} }}{{{\text{W}}_{1} }} \times 100$$

where W_0_ is the weight of the empty centrifuge tube, W_1_ is the weight of the centrifuge tube with gel before centrifugation; W_2_ is the weight of the centrifuge tube after absorbing the water.

### FT-IR spectroscopy

FT-IR (INVENIO, Invenio Bruker, Germany) was used to characterize the structure of pectin. FT–IR spectra were recorded at a resolution of 4 cm^−1^ with 16 scans ranging from 400 to 4000 cm^−1^. The DE of pectin was analyzed by the previous method with FT-IR^29^. The ratio of absorption bond at 1745 cm^−1^ over the sum of the bonds at 1745 and 1628 cm^−1^ was related to the DE value of pectin (Eq. 5)^9^.

5 $${\text{DE}}\,(\% ) = \frac{{{\text{A}}_{1745} }}{{{\text{A}}_{1745} + {\text{A}}_{1628} }} \times 100$$

### XRD

Analysis of the pectin powder samples was performed using an X-ray diffractometer (ARL™ EQUINOX 100, Thermo Fisher Scientific, China). Scanning analysis of pectin powder samples from 5° to 60° diffraction angle (2θ) (step size 5° 2θ, time per step: 1 min).

## Antioxidant activities analysis of SP and FP

### DPPH radical-scavenging activity

The DPPH radical-scavenging activity of samples was determined by the method of Liu et al.^23^ with some modifications. A certain amount of DPPH radical was dissolved in ethanol at the concentration of 0.1 mg/mL. The pectin sample was dissolved in distilled water to produce solutions of different concentrations, to produce solutions of different concentrations. To each of these pectin solutions was added 3.0 mL of the described DPPH solution, and the resulting solution was shaken immediately and kept at room temperature and in the dark for 30 min. The absorbance of supernatant was measured against a blank (ethanol instead of the sample and DPPH solution) at 517 nm. The DPPH radical-scavenging activity was measured by the following Eq. (6):

6 $${\text{DPPH}}\,\,{\text{radical - scavenging}}\,\,{\text{activity}}\,(\% ) = \frac{{{\text{Abs}}_{{{\text{control}}}} - {\text{Abs}}_{{{\text{sample}}}} }}{{{\text{Abs}}_{{{\text{control}}}} }} \times 100$$

### ABTS radical-scavenging activity

For this experiment, ABTS diammonium salt (5 mL, 7 mmol/L) and potassium persulfate (5 mL, 2.45 mmol/L) were mixed to form ABTS free radicals and the resulting solution was incubated at 25 °C overnight in the dark. To attain the absorbance of 0.70 ± 0.05 at 734 nm, the dilution of ABTS solution was performed by adding PBS buffer solution. Samples were diluted to different concentration (1–5 mg/mL) and then 0.5 mL of the solution were added to 2.5 mL of ABTS solution^48^. All measurements reacted for 3 min, and were repeated three times. The free radical scavenging rate of ABTS free radicals was calculated by the following Eq. (7):

7 $${\text{ABTS radical - scavenging activity (\% )}} = \frac{{{\text{Abs}}_{{{\text{control}}}} {\text{ - Abs}}_{{{\text{sample}}}} }}{{{\text{Abs}}_{{{\text{control}}}} }} \times 100$$

## Conclusion

The process variables for ammonium oxalate extraction of fresh sunflower pectin were optimized by RSM, and finally light-colored LMP was directly obtained. The optimum extraction conditions were determined to be flowing: extraction time, 1.34 h; liquid–solid ratio, 15:1 mL/g; and ammonium oxalate concentration, 0.76% (w/v), which afforded a pectin yield of 7.36 ± 0.4%. The results of characterization shown freeze-drying damaged the molecular weight and structure of LMP, and led to unstable emulsification and oxidation resistance of FP. This research opened up a new way for pectin extraction, and an ideal drying process can be selected for different applications.
